# Effects of hemodialysis on blood fatty acids

**DOI:** 10.14814/phy2.14332

**Published:** 2020-01-24

**Authors:** Benjamin Gollasch, Inci Dogan, Michael Rothe, Maik Gollasch, Friedrich C. Luft

**Affiliations:** ^1^ Experimental and Clinical Research Center (ECRC) A joint institution of the Charité Medical Faculty and Max Delbrück Center (MDC) for Molecular Medicine Berlin Germany; ^2^ HELIOS Klinikum Berlin‐Buch Berlin Germany; ^3^ LIPIDOMIX GmbH Berlin Germany; ^4^ Universitätsmedizin Greifswald Klinik für Geriatrie Greifswald Germany

**Keywords:** chronic kidney disease, dialysis, erythrocytes, exercise, fatty acids, lipidomics

## Abstract

Omega‐3 (n‐3) fatty acids have beneficial cardiovascular effects, perhaps also in chronic kidney disease (CKD) patients. A low omega‐3 index is an independent cardiovascular risk factor in end‐stage renal disease (ESRD) dialysis patients. However, the plasma measurements invariably ignore circulating blood cells, including the preponderant erythrocytes (RBCs). We measured fatty acids (HPLC‐MS lipidomics) in all components of the circulating blood, since RBC n‐3 fatty acid status has been linked to cardiovascular disease and mortality. We studied 15 healthy persons and 15 CKD patients undergoing regular hemodialysis treatments. While total fatty acid levels differed significantly in RBCs from healthy controls and CKD patients, the hemodialysis treatment had no effect on plasma or RBC fatty acid levels. No changes occurred in the percentage of eicosapentaenoic acid (C20:5 n‐3, EPA) and docosahexaenoic acid (C22:6 n‐3; DHA) (omega‐3 quotient) in RBC membrane fatty acids. Nonetheless, hemodialysis treatments increased plasma levels of various total fatty acids, namely C12:0, C14:0, C16:0, C20:2 n‐6, C20:4 n‐6, and C22:6 n‐3 (DHA), while plasma levels of free fatty acids were unchanged. These data suggest that despite significant changes in fatty acids signatures between healthy persons and CKD patients, hemodialysis does not alter RBC n‐3 fatty acid status, including the omega‐3 quotient. The dialysis treatment per se does not appear to be responsible for a lower omega‐3 index in CKD patients.

## Graphical abstract

Omega‐3 (n‐3) fatty acids have beneficial cardiovascular effects, perhaps also in chronic kidney disease (CKD) patients. We measured fatty acids (HPLC‐MS lipidomics) in all components of the circulating blood. Our data demonstrate significant changes in fatty acid signatures between healthy persons and CKD patients. However, dialysis treatment per se does not appear to be responsible for a lower omega‐3 index in CKD patients.

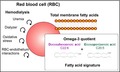

**Abbreviations**
C12:0: lauric acid
C14:0: myristic acid
C14:1: myristolein acid
C16:0: palmitic acid
C16:1: palmitoleic acid
C18:0: stearic acid
C18:1 cis: oleic acid (OA)
C18:1 trans: elaidic acid
C18:2: linoleic acid
C18:3 n-3: alpha-linolenic acid (ALA)
C18:3 n-6: gamma-linolenic acid (LA)
C20:0: arachidonic acid, eicosanoic acid
C20:1: eicosenoic acid
C20:2 n-6: eicosadienoic acid omega 6
C20:3: eicosatrienoic acid
C20:4 n-3: eicosatetraenoic acid omega-3
C20:4 n-6: eicosatetraenoic acid omega-6
C20:5 n-3: eicosapentaenoic acid (EPA)
C22:0: behenic acid, docosanoic acid
C22:1 n-9: erucic acid
C22:2 n-6: docosadienoic acid omega-6
C22:5 n-3: docosapentaenoic acid (DPA) omega-3
C22:5 n-6: docosapentaenoic acid (DPA) omega-6
C22:6 n-3: docosahexaenoic acid (DHA)
C24:1 n-9: nervonic acid

## 1 INTRODUCTION

Chronic kidney disease (CKD) is a leading risk factor for cardiovascular and all‐cause mortality. The 5‐year mortality of end‐stage renal disease (ESRD) hemodialysis patients approaches 50% (McGill et al., 2019), and most of these deaths are related to cardiovascular disease (CVD) (Felasa, 2012), making ESRD a catastrophic risk factor (Luft, 2000). Dietary omega‐3 (n‐3) fatty acid intake is associated with a reduced CVD risk (Harris, Kris‐Etherton, & Harris, 2008; Huang, Frohlich, & Ignaszewski, 2011; InterAct Consortium et al., 2011). Erythrocyte red‐blood‐cell (RBC) n‐3 fatty acid status is related to cardiac arrhythmias, myocardial infarction, and sudden cardiac death (Bucher, Hengstler, Schindler, & Meier, 2002).

The two most important n‐3 polyunsaturated fatty acids (PUFA) are eicosapentaenoic acid (C20:5 n‐3, EPA) and docosahexaenoic acid (C22:6 n‐3, DHA). However, the impact of the individual fatty acids for the predicting risk is not clearly elucidated. In recent randomized, double‐blind, placebo‐controlled trials, dietary n‐3 fatty acid supplementation (3–6 g daily) improved coronary atherosclerosis (Schacky, Angerer, Kothny, Theisen, & Mudra, 1999), but had (1 g daily) no cardiovascular benefit in initially healthy adults or in diabetic patients (ASCEND Study Collaborative Group et al., 2018; Manson et al., 2019). Two more recent large‐scale trials showed that dietary EPA (C20:5 n‐3) (4 g daily) is effective for prevention of major coronary events in hypercholesterolemic patients (Yokoyama et al., 2007) and reduces cardiovascular events in patients with established CVD, including in those with diabetes mellitus and other risk factors (Bhatt et al., 2018). Current evidence suggests that circulating n‐3 PUFA is also associated with lower risk of cardiovascular events and mortality in ESRD patients undergoing regular hemodialysis treatment (Chowdhury et al., 2014; Khor et al., 2018).

**Figure 1:**
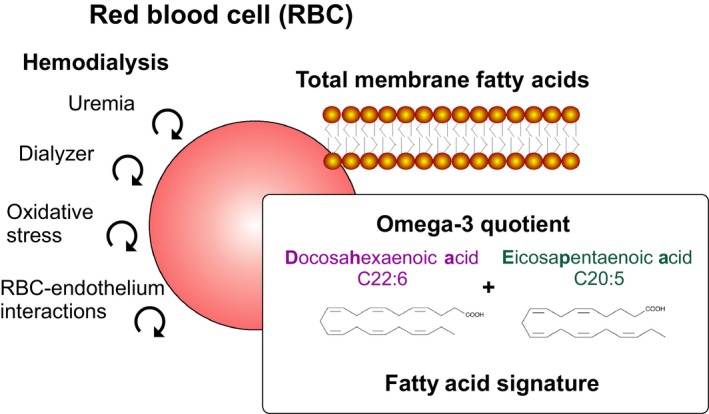
Schematic illustration of the hypothetic influence of hemodialysis associated with blood passing through the dialyzer, oxidative stress, uremia and red blood cell (RBC)–endothelial interactions affecting relative EPA + DHA content in RBC, that is, omega‐3 quotient, which is the percentage of EPA + DHA in red cell fatty acid lipids

Omega‐3 PUFA diets affect the RBC membrane fatty acid composition (Popp‐Snijders, Schouten, van Blitterswijk, & van der Veen, 1986). For example, a daily fish‐oil concentrate supplement, providing 3 g of n‐3 PUFA, increases incorporation of C20:5 n‐3 into RBCs. This effect occurs at the expense of C18:2 n‐6 fatty acids, indicating a possible role for RBC fatty acid profiles, that is, signatures, involved in the cardiovascular effects (Cartwright, Pockley, Galloway, Greaves, & Preston, 1985). The n‐6 fatty acid, C18:2, and the n‐3 fatty acids, C18:2, C20:5 (EPA), and C22:6 (DHA), collectively protect against coronary artery disease (Wijendran & Hayes, 2004). Patients with low RBC n‐3 and n‐6 fatty acid values, namely C16:1 (palmitoleic acid) and C18:0 (stearic acid), have an increased risk of acute coronary syndromes (Shearer, Pottala, Spertus, & Harris, 2009). Furthermore, C20:5 n‐3 (EPA) and C22:6 n‐3 (DHA) improve endothelial function, lower systemic blood pressure, and have favorable effects on platelet function (Wijendran & Hayes, 2004). A low omega‐3 index (the percentage of EPA + DHA in red‐blood‐cell lipids) independently increases cardiovascular disease risk and mortality (Kim, Lee, & An, 2018; Kleber, Delgado, Lorkowski, & Marz, 2016a; Kleber, Delgado, Lorkowski, Marz, & Schacky, 2016b; Schacky, 2015; Thuppal et al., 2017). RBC fatty acid status is similarly important in patients with CKD and ESRD (Khor et al., 2018; Kim et al., 2018). However, the impact of fatty acid measurements in the blood or RBC membranes for the prediction of CVD and mortality has not been previously elucidated. Whether or not the hemodialysis treatment itself affects blood or RBC membrane fatty acids is unknown. Conceivably, the hemodialysis treatment per se has a counterproductive effect on cardiovascular risk. We tested the hypothesis that hemodialysis affects RBC omega‐3 quotients and fatty acid signatures (Figure 1).

## 2 METHODS

The Charité University Medicine institutional review board on the use of humans in research approved the study, and written informed consent was obtained. The study was duly registered: ClinicalTrials.gov, Identifier: NCT03857984; institutional review board, EA2/113/08. Recruitment was primarily *via* person‐to‐person interview. Prior to participation in the study, 15 healthy volunteers (6 male and 9 female) and 15 CKD patients (7 male and 8 female) undergoing regular hemodialysis treatment signed informed consent forms which outlined the treatments to be taken and the possible risks involved (Table 1). We included patients who had a history of renal failure and requiring hemodialysis/hemofiltration, aged ≥18 years, and were able to consent in writing. Exclusion criteria for healthy control group were age <18 years, chronic illness requiring specific medications (such as corticosteroids, for instance), pregnancy, inability to follow simple instructions, or debilitating medical conditions. No healthy control subjects were taking medications. The patients included in the group CKD were diagnosed for the following conditions: diabetes mellitus (4 patients), hypertension (3 patients), membranous glomerulonephritis (2 patients), ADPKD (autosomal‐dominant polycystic kidney disease) (1 patient), other, or unknown (5 patients).

**Table 1:** Characteristics of hemodialysis (HD) patients and control subjects

	HD patients (*n* = 15)	Controls (*n* = 15)	Statistical significance
Age (*n*)	50 ± 18	47 ± 12	*p* > .05
Sex			
Male (*n*)	6	7	
Female (*n*)	9	8	
Body mass index (kg/m^2^)	24.8 ± 3.4	24.7 ± 4.6	*p* > .05
Race (*n*)			
	Caucasian = 14	Caucasian = 14	
	Black = 1	Asian = 1	
Cause of end‐stage renal disease			
Diabetes (*n*)	4		
Hypertension (*n*)	3		
Membranous GN (*n*)	2		
ADPKD (*n*)	1		
Other or unknown	5		
Complications			
Cardiovascular (*n*)	2		
Cerebrovascular (*n*)	1		
PAD (*n*)	3		

Note

Data are presented as mean ± *SD* or frequencies.

Abbreviations: GN, glomerulonephritis; *n*, number of patients; PAD, peripheral artery disease.

Venous blood was collected in each healthy subject by subcutaneous arm vein puncture in the sitting position. In the group of dialyzed patients (CKD group), all the blood samples were collected on the fistula arm right before beginning of the dialysis (starting of the HD, pre‐HD) and at the end of the dialysis (5–15 min before termination, post‐HD). Patients underwent thrice‐weekly dialysis, which lasted from 3 hr 45 min to 5 hr, based on high flux AK 200 dialyzers (Gambro GmbH). All samples were analyzed for RBC fatty acids status and plasma fatty acids. RBCs were separated from EDTA blood by centrifugation, and fatty acids in RBCs or plasma were determined by high‐performance liquid chromatography‐mass spectrometry (HPLC‐MS) described in Fischer et al., (2014); Gollasch, Dogan, Rothe, Gollasch, and Luft (2019).

We performed sample size calculation for a difference in means in omega‐3 quotients. We found that our study would require a sample size of 9 (number of pairs) to achieve a power of 80% and a level of significance of 5% (two sided), for detecting a mean of the differences of 2.1 (Harris, Del Gobbo, and Tintle (2017)) between pairs, assuming the standard deviation of the differences to be 1.7 (Fischer et al., 2014; Gollasch et al., 2019). A 2.1% increase in omega‐3 index is associated with a 15% risk reduction for fatal coronary heart disease relative to the mean level (Harris et al., 2017). Thus, in our sample size calculation, statistical significance and clinical relevance both were taken into account.

Descriptive statistics were calculated, and variables were examined for meeting assumptions of normal distribution without skewness and kurtosis. We used the Shapiro–Wilk test to determine whether the data were normally distributed. In order to determine statistical significance, two‐tailed *t* test or Mann–Whitney test were used to compare values of CKD versus control groups. Homogeneity of variances was asserted using Levene's test. Paired *t* test or paired Wilcoxon test was used to compare pre‐HD versus post‐HD values. The .05 level of significance (*p*) was chosen. All data are presented as mean ± *SD*. All statistical analyses were performed using SPSS Statistics software (IBM Corporation) or All‐Therapy statistics beta (AICBT Ltd).

## 3 RESULTS

### 3.1 Clinical characteristics

The clinical and demographic characteristics of the patients and control subjects (Table 1) show that age between ESRD patients and the healthy subjects was not different. The body mass indices between the two groups were also not different. The ESRD patients had diabetes mellitus, hypertension, membranous glomerulonephritis, autosomal‐dominant polycystic kidney disease (ADPKD), and other or unknown disease, as underlying causes. Major cardiovascular complications including cardiovascular and cerebrovascular events, and peripheral artery disease are given. Subjects were Caucasians, with the exception of one Black and one Asian subject in each group.

### 3.2 Fatty acid status comparing controls to ESRD patients

We first determined the RBC fatty acid status in healthy control subjects and compared the results with ESRD patients (Table 2). The data demonstrate decreased incorporation of total EPA (C20:5 n‐3), C14:1 n‐5, C18:3 n‐6, C20:3 n‐6, and C22:1 n‐9 and increased incorporation of total C24:1 n‐9 levels into RBCs of CKD patients compared to control subject (Table 2A). Moreover, the patients showed also lower levels of total C18:3 n‐6 and C20:3 n‐6 fatty acids in RBCs. These differences occurred at the expense of increased levels of total C22:1 n‐9 and C24:1 n‐9 fatty acids, because total RBC fatty acids were unchanged (Table 3A). No changes occurred in the levels of total C12:0, C14:0, C14:1 n‐5, C16:0, C16:1 n‐7, C18:0, C18:1 n‐9, C18:2 n‐6, C18:3 n‐3, C20:1 n‐9, C20:2 n‐6, C20:4 n‐6, C22:2 n‐6, C22:5 n‐3, C22:5 n‐6, and C22:6 n‐3 fatty acids in RBCs. The results on total fatty acids in plasma of control subjects and CKD patients are summarized (Table 2B). The data demonstrate decreased levels of total C18:2 n‐6, C20:3 n‐6, C20:4 n‐6, C20:5 n‐3 (EPA), and C22:5 n‐6 in plasma of CKD patients compared to control subject. No differences were observed in the levels of total C12:0, C14:0, C14:1 n‐5, C16:0, C16:1 n‐7, C18:0, C18:1 n‐9, C18:3 n‐3, C18:3 n‐6, C20:1 n‐9, C20:2 n‐6, C22:1 n‐9, C22:2 n‐6, C22:5 n‐3, C22:6 n‐3, and C24:1 n‐9 fatty acids in plasma. The levels of most free fatty acids (C12:0–C24:1 n‐9) measured were increased in plasma of CKD patients compared to healthy controls (Table 2C). The same was true for free fatty acids in RBCs (Table 2D).

**Table 2:** Comparison of blood fatty acids between control subjects versus hemodialysis (HD) patients before hemodialysis (*n* = 15 each)

(A) Total fatty acids in erythrocytes (RBC)			
Amount µg/g	Control (mean ± *SD*)	HD (mean ± *SD*)	*p* value *t* test (^#^Mann–Whitney test)
C12:0	4.544 ± 1.847	6.033 ± 2.598	.089^#^
C14:0	19.704 ± 8.466	21.248 ± 17.289	.486^#^
C14:1 n−5	0.923 ± 0.772	0.661 ± 1.317	.033^#^
C16:0	147.795 ± 33.600	151.751 ± 46.741	.792
C16:1 n−7	12.089 ± 5.733	10.718 ± 8.042	.187^#^
C18:0	139.167 ± 40.465	147.867 ± 49.442	.602
C18:1 n−9 (cis)	189.604 ± 29.953	202.867 ± 75.846	.967^#^
C18:1n−9 (trans)	5.718 ± 1.744	7.199 ± 4.501	.250
C18:2 n−6	151.432 ± 21.535	126.833 ± 46.121	.072
C18:3 n−3	6.870 ± 2.754	6.097 ± 3.448	.305^#^
C18:3 n−6	4.127 ± 1.855	2.670 ± 1.847	.016^#^
C20:1 n−9	2.703 ± 0.632	3.307 ± 1.407	.145
C20:2 n−6	1.746 ± 0.308	1.762 ± 0.471	.935^#^
C20:3 n−6	11.726 ± 2.078	9.600 ± 3.360	.046
C20:4 n−6	144.244 ± 17.584	133.665 ± 33.127	.284
**C20:5 n−3**	19.538 ± 9.789	18.558 ± 24.782	.021^#^
C22:1 n−9	1.132 ± 0.372	1.971 ± 1.461	.026^#^
C22:2 n−6	0.260 ± 0.097	0.307 ± 0.162	.348
C22:5 n−3	15.802 ± 2.565	17.445 ± 7.140	.413
C22:5 n−6	3.624 ± 0.664	3.077 ± 1.002	.089
**C22:6 n−3**	76.838 ± 25.022	75.603 ± 35.053	.461^#^
C24:1 n−9	2.360 ± 1.149	4.799 ± 2.767	.002^#^

(B) Total fatty acids in plasma			
Amount µg/ml	Control (mean ± *SD*)	HD (mean ± *SD*)	*p* value *t* test (^#^Mann–Whitney test)
C12:0	13.22 ± 11.31	12.00 ± 10.17	.624^#^
C14:0	48.00 ± 33.46	43.91 ± 45.24	.161^#^
C14:1 n−5	3.28 ± 3.63	2.99 ± 5.11	.367^#^
C16:0	220.18 ± 59.31	190.31 ± 80.84	.081^#^
C16:1 n−7	21.68 ± 9.68	20.30 ± 15.16	.412^#^
C18:0	146.92 ± 34.71	137.83 ± 48.88	.305^#^
C18:1 n−9 (cis)	281.00 ± 88.78	281.24 ± 158.52	.595^#^
C18:1n−9 (trans)	7.46 ± 3.20	6.34 + 6.32	.061^#^
C18:2 n−6	214.23 ± 44.62	192.13 ± 110.50	.037^#^
C18:3 n−3	13.63 ± 8.09	11.68 ± 5.68	.595^#^
C18:3 n−6	10.42 ± 6.19	9.04 ± 4.42	.624^#^
C20:1 n−9	3.47 ± 1.17	3.86 ± 2.14	.935^#^
C20:2 n−6	2.22 ± 0.78	2.15 ± 1.13	.486^#^
C20:3 n−6	13.98 ± 4.18	9.36 ± 5.00	.003^#^
C20:4 n−6	104.63 ± 27.37	70.47 ± 31.09	.001^#^
**C20:5 n−3**	24.39 ± 13.10	19.48 ± 27.18	.023^#^
C22:1 n−9	2.09 ± 0.61	2.06 ± 0.74	.927
C22:2 n−6	0.00 ± 0.00	0.00 ± 0.00	n/a
C22:5 n−3	5.80 ± 1.74	4.80 ± 1.49	.102
C22:5 n−6	1.80 ± 0.70	1.23 ± 0.68	.021^#^
**C22:6 n−3**	51.18 ± 16.55	41.73 ± 27.37	.050^#^
C24:1 n−9	2.22 ± 0.65	2.39 ± 0.68	.497

(C) Free fatty acids in plasma			
Amount µg/ml	Control (mean ± *SD*)	HD (mean ± *SD*)	*p* value *t* test (^#^Mann–Whitney test)
C12:0	1.203 ± 0,353	2.196 ± 1.419	.001^#^
C14:0	2.696 ± 1.054	4.160 ± 1.239	.005^#^
C14:1 n−5	0.205 ± 0.226	0.308 ± 0.153	.048^#^
C16:0	24.484 ± 4.204	29.251 ± 6.602	.038
C16:1 n−7	2.185 ± 2.329	4.622 ± 2.807	.017^#^
C18:0	16.525 ± 2.152	17.919 ± 3.449	.226
C18:1 n−9 (cis)	16.385 ± 9.252	28.061 ± 13.685	.015
C18:1n−9 (trans)	n.d.	n.d.	—
C18:2 n−6	5.681 ± 3.703	15.252 ± 14.938	.043^#^
C18:3 n−3	0.842 ± 0.658	2.234 ± 3.117	.043^#^
C18:3 n−6	0.127 ± 0.092	0.455 ± 0.505	.014^#^
C20:0	0.660 ± 0.113	0.848 ± 0.201	.008
C20:1 n−9	0.670 ± 0.378	1.501 ± 0.884	.006
C20:2 n−6	0.323 ± 0.069	0.527 ± 0.295	.012^#^
C20:3 n−6	0.082 ± 0.481	0.214 ± 0.219	.094^#^
C20:4 n−3	0.010 ± 0.056	0.029 ± 0.049	.017^#^
C20:4 n−6	1.049 ± 0.450	1.146 ± 0.846	.715
**C20:5 n−3**	0.640 ± 0.057	0.826 ± 2.394	.830^#^
C22:0	0.053 ± 0.020	0.167 ± 0.149	<.001^#^
C22:1 n−9	0.066 ± 0.450	0.232 ± 0.337	.076^#^
C22:2 n−6	n.d.	n.d.	—
C22:5 n−3	0.127 ± 0.590	0.459 ± 0.898	.012^#^
C22:5 n−6	0.066 ± 0.020	0.118 ± 0.121	.280^#^
**C22:6 n−3**	0.345 + 0.213	1.271 ± 3.089	.756^#^
C24:1 n−9	0.072 ± 0.018	0.117 ± 0.067	.036

(D) Free fatty acids in erythrocytes			
Amount µg/g	Control (mean ± *SD*)	HD (mean ± *SD*)	*p* value *t* test (^#^Mann–Whitney test)
C12:0	0.035 ± 0.014	0.069 ± 0.054	<.001^#^
C14:0	0.200 ± 0.096	0.393 ± 0.133	<.001^#^
C14:1 n−5	0.008 ± 0.010	0.019 ± 0.010	.002^#^
C16:0	1.797 ± 0.213	3.346 ± 0.425	<.001
C16:1 n−7	0.199 ± 0.210	0.460 ± 0.257	.003^#^
C18:0	4.951 ± 0.817	8.431 ± 1.380	<.001
C18:1 n−9 (cis)	1.065 ± 0.489	2.863 ± 1.262	<.001
C18:1n−9 (trans)	0.056 ± 0.043	0.109 ± 0.060	.002^#^
C18:2 n−6	0.388 ± 0.217	1.355 ± 1.414	<.001^#^
C18:3 n−3	0.082 ± 0.058	0.302 ± 0.384	<.001^#^
C18:3 n−6	0.012 ± 0.007	0.038 ± 0.048	.008^#^
C20:1 n−9	0.041 ± 0.033	0.084 ± 0.053	.003^#^
C20:2 n−6	0.018 ± 0.012	0.055 ± 0.046	<.001^#^
C20:3 n−6	0.015 ± 0.007	0.034 ± 0.025	<.001^#^
C20:4 n−6	0.221 ± 0.065	0.374 ± 0.206	.007^#^
**C20:5 n−3**	0.037 ± 0.019	0.168 ± 0.492	.367^#^
C22:1 n−9	0.007 ± 0.003	0.034 ± 0.046	<.001^#^
C22:2 n−6	0.001 ± 0.001	0.003 ± 0.003	<.001^#^
C22:5 n−3	0.413 ± 0.212	2.107 ± 3.741	<.001^#^
C22:5 n−6	0.201 ± 0.114	0.530 ± 0.624	.006^#^
**C22:6 n−3**	0.051 ± 0.025	0.222 ± 0.456	.002^#^
C24:1 n−9	0.361 ± 0.166	0.955 ± 0.508	<.001

Note

Bold indicates major fatty acids critical for the omega 3‐ quotient (Figure 1).

**Table 3:** Omega‐3 quotients in control subjects versus hemodialysis (HD) patients before hemodialysis (*n* = 15 each)

(A) Omega−3 quotient of RBC total fatty acids			
Fatty acid (µg/g)	Control (Mean ± *SD*)	ESRD (Mean ± *SD*)	*t* test, *p* value (^#^Mann–Whitney test)
C20:5 n−3 (EPA) + C22:6 n−3 (DHA)	96.377 ± 28.078	94.161 ± 54.387	.233^#^
Total fatty acids in RBCs	961.952 ± 139.688	954.043 ± 234.962	.912
C20:5 n−3 (EPA) ± C22:6 n−3 (DHA)]/ total fatty acids in RBCs	0.100 ± 0.021	0.100 ± 0.056	.325^#^

(B) Omega−3 quotient of RBC free fatty acids			
Fatty acid (µg/g)	Control (Mean ± *SD*)	ESRD (Mean ± *SD*)	*t* test, *p* value (^#^Mann–Whitney test)
C20:5 n−3 (EPA) + C22:6 n−3 (DHA)	0.088 ± 0.031	0.389 ± 0.951	.011^#^
Total free fatty acids	10.16 ± 1.93	21.96 ± 9.30	<.001^#^
[C20:5 n−3 (EPA) + C22:6 n−3 (DHA)]/ Total free fatty acids	0.0087 ± 0.0027	0.0114 ± 0.0178	.202^#^

We next inspected the omega‐3 quotients in control subjects and ESRD patients (Table 3). Within RBC, the omega‐3 quotient of total fatty acids was not different (Table 3A). No changes occurred in the omega‐3 quotient, that is, the percentage of total eicosapentaenoic acid (C20:5 n‐3; EPA) and docosahexaenoic acid (C22:6 n‐3; DHA) in RBC fatty acids in the RBC membrane. However, the omega‐3 quotient of free fatty acids within RBC showed increased values for C20:5 n‐3 (EPA) + C22:6 n‐3 (DHA) and increased total free fatty acids in ESRD patients, compared to controls (Table 3B). The omega‐3 quotient of free fatty acids in erythrocytes of CKD patients did not differ from the quotient measured in control subjects, despite higher free C20:5 n‐3 (EPA) plus C22:6 n‐3 (DHA) levels and lower total levels of free fatty acids (C12:0–C24:1 n‐9) in the RBCs of CKD patients.

### 3.3 Effects of hemodialysis

We inspected the effects of hemodialysis treatment on blood fatty acids in the CKD patients before (pre‐HD) hemodialysis and at cessation (post‐HD) of hemodialysis in the 15 ESRD patients (Table 4). The total fatty acids in RBCs were not significantly affected (except for C18:2, which showed a slight decrease after hemodialysis) (Tables 4A). The total fatty acids in plasma showed a significant increase in C12:0, C14:0, C16:0, and C24:1 or decrease in C18:0, C20:2, and C20:4 (Table 4B). The free fatty acids in plasma were not affected by the hemodialysis treatment (Table 4C). Similarly, the free fatty acid concentrations within RBCs were not affected (Table 4D). Since both circulating total and free C18:3 n‐6, C20:3 n‐6, C20:5 n‐3 (EPA), C22:1 n‐9, and C24:1 n‐9 fatty acids in plasma were not affected by hemodialysis, the observed changes in total RBC C18:3 n‐6, C20:3 n‐6, C20:3 n‐6, C20:5 n‐3 (EPA), C22:1 n‐9, and C24:1 n‐9 levels in ESRD patients are unlikely caused by uptake into "exchangeable" or "reversibly bound" free fatty acid pools in the erythrocytes in response to individual hemodialysis treatments.

**Table 4:** Effects of hemodialysis treatment on blood fatty acids in the CKD patients before (pre‐HD) hemodialysis and at cessation (post‐HD) of hemodialysis (*n* = 15 each)

(A) Total fatty acids in erythrocytes			
Amount µg/g	Pre‐HD (mean ± *SD*)	Post‐HD (mean ± *SD*)	*p* value Paired *t* test (^#^paired Wilcoxon test)
C12:0	6.033 ± 2.598	6.047 ± 3.472	.776^#^
C14:0	21.248 ± 17.289	20.794 ± 18.874	.910^#^
C14:1 n−5	0.661 ± 1.317	0.654 ± 1.409	.445^#^
C16:0	151.751 ± 46.741	142.066 ± 46.454	.456
C16:1 n−7	10.718 ± 8.042	8.763 ± 5.835	.061^#^
C18:0	147.867 ± 49.442	139.942 ± 53.184	.622
C18:1 n−9 (cis)	202.867 ± 75.846	190.543 ± 60.520	.233^#^
C18:1n−9 (trans)	7.199 ± 4.501	6.119 ± 4.864	.156^#^
C18:2 n−6	126.833 ± 46.121	111.659 ± 28.970	.045
C18:3 n−3	6.097 ± 3.448	6.178 ± 4.241	.820^#^
C18:3 n−6	2.670 ± 1.847	2.132 ± 1.039	.112^#^
C20:1 n−9	3.307 ± 1.407	3.006 ± 0.995	.405
C20:2 n−6	1.762 ± 0.471	1.698 ± 0.412	.609^#^
C20:3 n−6	9.600 ± 3.360	9.341 ± 3.521	.394^#^
C20:4 n−6	133.665 ± 33.127	137.584 ± 26.824	.487
**C20:5 n−3**	18.558 ± 24.782	19.364 ± 26.036	.112^#^
C22:1 n−9	1.971 ± 1.461	1.395 ± 0.758	.256^#^
C22:2 n−6	0.307 ± 0.162	0.303 ± 0.202	.955^#^
C22:5 n−3	17.445 ± 7.140	18.699 ± 7.148	.313
C22:5 n−6	3.077 ± 1.002	3.231 ± 0.937	.435
**C22:6 n−3**	75.603 ± 35.053	80.789 ± 37.475	.088^#^
C24:1 n−9	4.799 ± 2.767	4.680 ± 3.153	.890

(B) Total fatty acids in plasma			
Amount µg/ml	Pre‐HD (mean ± *SD*)	Post‐HD (mean ± *SD*)	*p* value Paired *t* test (^#^paired Wilcoxon test)
C12:0	12.00 ± 10.17	16.26 ± 15.70	.006^#^
C14:0	43.91 ± 45.24	55.09 ± 63.93	.048^#^
C14:1 n−5	2.99 ± 5.11	3.24 ± 5.25	.422^#^
C16:0	190.31 ± 80.84	212.30 ± 77.50	.016^#^
C16:1 n−7	20.30 ± 15.16	19.85 ± 12.12	.730^#^
C18:0	137.83 ± 48.88	151.93 ± 46.05	.026^#^
C18:1 n−9 (cis)	281.24 ± 158.52	316.19 ± 139.41	.096^#^
C18:1n−9 (trans)	6.34 ± 6.32	7.71 ± 9.91	.096^#^
C18:2 n−6	192.13 ± 110.50	218.67 ± 109.55	.158^#^
C18:3 n−3	11.68 ± 5.68	14.21 ± 11.07	.397^#^
C18:3 n−6	9.04 ± 4.42	10.98 ± 8.55	.397^#^
C20:1 n−9	3.86 ± 2.14	4.31 ± 1.85	.124^#^
C20:2 n−6	2.15 ± 1.13	2.32 ± 1.03	.041^#^
C20:3 n−6	9.36 ± 5.00	10.00 ± 5.15	.109^#^
C20:4 n−6	70.47 ± 31.09	77.44 ± 28.49	.035^#^
**C20:5 n−3**	19.48 ± 27.18	38.53 ± 96.14	.084
C22:1 n−9	2.06 ± 0.74	2.63 ± 1.35	.074
C22:2 n−6	0.00 ± 0.00	0.00 ± 0.00	n/a
C22:5 n−3	4.80 ± 1.49	5.66 ± 3.04	.140
C22:5 n−6	1.23 ± 0.68	1.41 ± 0.69	.397
**C22:6 n−3**	41.73 ± 27.37	50.37 ± 45.26	.433
C24:1 n−9	2.39 ± 0.68	2.79 ± 1.00	.022

(C) Free fatty acids in plasma			
Amount µg/ml	Pre‐HD (mean ± *SD*)	Post‐HD (mean ± *SD*)	*p* value Paired *t* test (#paired Wilcoxon Test)
C12:0	2.196 ± 1.419	2.398 ± 1.101	.937^#^
C14:0	4.160 ± 1.239	5.317 ± 2.810	.583^#^
C14:1 n−5	0.308 ± 0.153	0.574 ± 0.498	.187
C16:0	29.251 ± 6.602	30.510 ± 5.052	.994
C16:1 n−7	4.622 ± 2.807	5.093 ± 3.265	.974
C18:0	17.919 ± 3.449	18.521 ± 2.676	.960
C18:1 n−9 (cis)	28.061 ± 13.685	31.285 ± 10.839	.899
C18:1 n−9 (trans)	n.d.	n.d.	—
C18:2 n−6	15.252 ± 14.938	14.577 ± 8.750	.814^#^
C18:3 n−3	2.234 ± 3.117	2.411 ± 2.138	.695^#^
C18:3 n−6	0.455 ± 0.505	0.393 ± 0.243	.754^#^
C20:0	0.848 ± 0.201	0.926 ± 0.173	.638
C20:1 n−9	1.501 ± 0.884	1.835 ± 0.956	.346
C20:2 n−6	0.527 ± 0.295	0.499 ± 0.198	.814^#^
C20:3 n−6	0.214 ± 0.219	0.194 ± 0.119	.875^#^
C20:4 n−3	0.029 ± 0.049	0.034 ± 0.046	.875^#^
C20:4 n−6	1.146 ± 0.846	1.257 ± 0.703	.875^#^
**C20:5 n−3**	0.826 ± 2.394	0.694 ± 1.840	.433^#^
C22:0	0.167 ± 0.149	0.172 ± 0.140	1.000^#^
C22:1 n−9	0.232 ± 0.337	0.269 ± 0.336	.209^#^
C22:2 n−6	n.d.	n.d.	—
C22:5 n−3	0.459 ± 0.898	0.423 ± 0.582	.875^#^
C22:5 n−6	0.118 ± 0.121	0.110 ± 0.770	.814^#^
**C22:6 n−3**	1.271 ± 3.089	1.155 ± 2.285	.347^#^
C24:1 n−9	0.117 ± 0.067	0.132 ± 0.090	.395

(D) Free fatty acid in erythrocytes			
Amount µg/g	Pre‐HD (mean ± *SD*)	Post‐HD (mean ± *SD*)	*p* value Paired *t* test (^#^paired Wilcoxon Test)
C12:0	0.082 ± 0.054	0.094 ± 0.068	.496^#^
C14:0	0.394 ± 0.133	0.490 ± 0.308	.281^#^
C14:1 n−5	0.019 ± 0.010	0.032 ± 0.031	.191^#^
C16:0	3.346 ± 0.425	3.519 ± 0.401	.196
C16:1 n−7	0.460 ± 0.257	0.552 ± 0.387	.357
C18:0	8.431 ± 1.380	8.716 ± 1.094	.484
C18:1 n−9 (cis)	2.863 ± 1.262	3.313 ± 1.274	.284
C18:1n−9 (trans)	0.109 ± 0.060	0.170 ± 0.178	.053^#^
C18:2 n−6	1.355 ± 1.414	1.279 ± 0.658	.609^#^
C18:3 n−3	0.302 ± 0.384	0.365 ± 0.352	.394^#^
C18:3 n−6	0.038 ± 0.048	0.033 ± 0.019	.609^#^
C20:1 n−9	0.084 ± 0.053	0.116 ± 0.097	.191^#^
C20:2 n−6	0.055 ± 0.064	0.062 ± 0.044	.211^#^
C20:3 n−6	0.034 ± 0.025	0.035 ± 0.015	.191^#^
C20:4 n−6	0.374 ± 0.206	0.395 ± 0.124	.125^#^
**C20:5 n−3**	0.168 ± 0.498	0.116 ± 0.268	.125^#^
C22:1 n−9	0.034 ± 0.046	0.049 ± 0.059	.281^#^
C22:2 n−6	0.003 ± 0.003	0.003 ± 0.002	.112^#^
C22:5 n−3	2.107 ± 3.741	1.750 ± 1.432	.394^#^
C22:5 n−6	0.530 ± 0.624	0.424 ± 0.199	.532^#^
**C22:6 n−3**	0.222 ± 0.456	0.172 ± 0.211	.256^#^
C24:1 n−9	0.955 ± 0.508	1.166 ± 0.579	.082

Note

Bold indicates major fatty acids critical for the omega 3‐ quotient (Figure 1).

We also inspected the effects of hemodialysis on omega‐3 quotients (Table 5). C20:5 n‐3 (EPA) + C22:6 n‐3 (DHA) values in RBCs have not increased (*p* = .053), while no effects were also observed on total fatty acids or [C20:5 n‐3 (EPA) + C22:6 n‐3 (DHA)]/ total fatty acids in RBCs (Table 5A). Similarly, no effects were observed on the sum of RBC free fatty acids or omega‐3 quotient of free fatty acids in RBCs (Table 5B). Thus, no fatty acid‐level variations were found in RBCs in response to hemodialysis. Furthermore, no changes occurred also in the RBC omega‐3 quotient in response to hemodialysis. Together, the findings indicate that ESRD is associated with an altered RBC fatty acid status, that is, individual signature, which has no effect on the erythrocyte n‐3 fatty acid quotient. Moreover, hemodialysis treatment is insufficient to change the RBC fatty acid signature of ESRD patients.

**Table 5:** Effects of hemodialysis on omega‐3 quotients (*n* = 15 each)

A. Omega‐3 quotient of RBC total fatty acids in the CKD patients before (pre‐HD) hemodialysis and at cessation (post‐HD) of hemodialysis.			
Fatty acid (µg/g)	Pre‐HD (Mean ± *SD*)	Post‐HD (Mean ± *SD*)	Paired *t* test, *p* value (^#^paired Wilcoxon Test)
C20:5 n−3 (EPA) + C22:6 n−3 (DHA)	94.161 ± 54.387	100.153 ± 58.031	.053^#^
Total fatty acids in RBCs	954.043 ± 234.962	914.992 ± 207.009	.390
[C20:5 n−3 (EPA) ± C22:6 n−3 (DHA)]/ total fatty acids in RBCs	0.100 ± 0.056	0.108 ± 0.049	.088^#^

B. Omega‐3 quotient of plasma free fatty acids of the CKD patients before (pre‐HD) hemodialysis (HI) and at cessation (post‐HD) of hemodialysis (HII)			
Fatty acid (µg/ml)	Pre‐HD (Mean ± *SD*)	Post‐HD (Mean ± *SD*)	Paired *t* test, *p* value (^#^paired Wilcoxon Test)
C20:5 n−3 (EPA) + C22:6 n−3 (DHA)	0.389 ± 0.951	0.288 ± 0.474	.211^#^
Total free fatty acids	21.96 ± 9.30	22.85 ± 4.86	.281^#^
[C20:5 n−3 (EPA) + C22:6 n−3 (DHA)]/ Total free fatty acids	0.0114 ± 0.0178	0.0115 ± 0.0167	.733^#^

Of note, hemodialysis affected the total levels of various saturated and n‐6 fatty acids in plasma, which increased during hemodialysis. These fatty acids included C12:0, C14:0, C16:0, C20:2 n‐6, C20:4 n‐6, and C24:1 n‐9, which were detected as total fatty acids (Table 4B), but not in free state (Table 4C). In contrast, hemodialysis did not affect the levels of total C14:1 n‐5, C16:1 n‐7, C18:1 n‐9, C18:2 n‐6, C18:3 n‐3, C18:3 n‐6, C20:1 n‐9, C20:3 n‐6, C20:5 n‐3, C22:1 n‐9, C22:2 n‐6, C22:5 n‐3, C22:5 n‐6, and C22:6 n‐3 fatty acids in plasma.

## 4 DISCUSSION

We evaluated blood fatty acids in normal controls and ESRD patients. Also, we studied the effects of the hemodialysis treatment, which could be deleterious. Unique is the fact that we included the erythron in the analysis, rather than merely plasma values. The issue is important since the RBC mass (>40% of the circulating blood) is important to n‐3 homeostasis and metabolism. Since ESRD patients die on dialysis within 5 years, the hypothesis that the treatment (in‐and‐of‐itself) could be injurious seems reasonable. Our study investigated effects on n‐3 fatty acids, and we encompassed all of the components in the circulating blood.

To our knowledge, our study is the first study to assess the impact of single hemodialysis treatment on individual RBC fatty acids using large‐scale lipidomics. Although we did not confirm our hypothesis that RBC fatty acids, including the omega‐3 quotient, vary during hemodialysis, we observed significant differences in RBC fatty acid status, that is, specific fatty acid signatures, between ESRD patients and control subjects. However, the omega‐3 quotient did not vary between CKD patients and healthy volunteers. Finally, hemodialysis treatment did not induce increased mobilization of individual free fatty acids into plasma or erythrocytes, but caused greater rate of oxidation of total fatty acids, which accumulated in the circulating blood during hemodialysis.

### 4.1 Omega‐3 and omega‐6 fatty acids

A low omega‐3 index independently increases cardiovascular disease risk and mortality, perhaps also in CKD (Kim et al., (2018); Kleber et al., 2016b; Kleber et al., 2016a; Thuppal et al., 2017; Schacky, 2015). Consistent with a number of previous studies (for review see Khor et al. (2018)), we detected decreased RBC EPA (C20:5 n‐3) levels in our CKD patients, compared to the control subjects. These changes were paralleled by decreased RBC C18:3 n‐6 and C20:3 n‐6 levels and decreased plasma levels of C18:2 n‐6, C20:3 n‐6, C20:4 n‐6, C20:5 n‐3, and C22:5 n‐6, which is similar to previous findings (Dasgupta, Kenny, & Ahmad, 1990; Dessi et al., 2014; Friedman, Moe, Perkins, Li, & Watkins, 2006; Friedman et al., 2006; Gomez Dumm, Giammona, Touceda, & Raimondi, 2001; Pazda, Stepnowski, Sledzinski, Chmielewski, & Mika, 2017; Peuchant et al., 1994; Sertoglu et al., 2014; Sikorska‐Wisniewska et al., 2017; Yerlikaya, Mehmetoglu, Kurban, & Tonbul, 2011), for review see Khor et al. (2018)). However, the omega‐3 quotient did not vary between CKD patients and control subjects, which contrasts to numerous studies detecting a low omega‐3 index in CKD patients (for review see Khor et al. (2018)). Interestingly, we did not detect changes in C18:3 n‐3, which is inversely related to adiposity (Perng, Villamor, Mora‐Plazas, Marin, & Baylin, 2015). Together, the results indicate that there is an altered profile of n‐3/ n‐6 fatty acids in ESRD patients, which is confirmed by numerous clinical studies.

Dietary omega‐3 fatty acids modulate the profile of eicosanoids in humans primarily *via* the cytochrome P450 (CYP)‐epoxygenase pathway, which could mediate cardioprotective and vasodilatory effects of n‐3 fatty acids (Fischer et al., 2014). Recent results demonstrate that CYP enzymes efficiently convert C20:5 n‐3 (EPA) and C22:6 n‐3 (DHA) to bioactive epoxy and hydroxy metabolites that could mediate some of the beneficial cardiovascular effects of dietary n‐3 fatty acids (Arnold et al., 2010). Thus, pharmacological interventions targeting the CYP‐eicosanoid pathway could offer promising new options for cardiovascular disease risk and management. An alternative therapeutic approach is to focus on supplementation of individual fatty acids. As such, recent data show that dietary C20:5 n‐3 (EPA, 4 g daily, REDUCE‐IT trial) is effective for prevention of major coronary events in hypercholesterolemic patients (Yokoyama et al., 2007). Dietary C20:5 n‐3 supplementation is also effective for prevention of cardiovascular events in patients with established cardiovascular disease or with diabetes and other risk factors (Bhatt et al., 2018). Our results demonstrate that RBC fatty acids, including the omega‐3 quotient, do not vary during hemodialysis. The results are supported by studies, which did not use large‐scale lipidomics but rather focused on individual fatty acids and used smaller number of patients (Friedman, Siddiqui, & Watkins, 2008; Peuchant et al., 1994; Peuchant, Salles, Vallot, Wone, & Jensen, 1988; Taccone‐Gallucci et al., 1989). Our results support the concept that the omega‐3 quotient is strongly affected by diet, for example, C22:6 n‐3/C20:5 n‐3 fatty acid (DHA/EPA)‐rich diet (Begum, Belury, Burgess, & Peck, 2004; Fischer et al., 2014; Saifullah et al., 2007), but not hemodialysis treatment itself. The baseline differences in RBC C20:5 n‐3 (EPA), C18:3 n‐6, and C20:3 n‐6 levels observed in CKD patients may contribute to increased cardiovascular risk in ESRD.

### 4.2 Omega‐9 fatty acids

High levels of omega‐9 (n‐9) monounsaturated fatty acids, C18:1 n‐9 cis, C20:1 n‐9, and C24:1 n‐9 in RBCs have been associated with increased cardiovascular mortality in the Ludwigshafen Risk and Cardiovascular Health Study (Delgado et al., 2017). High levels of C16:0, C16:1 n‐7, C18:1 n‐9, and C18:3 n‐3 in RBCs were also associated with increased risk of sudden cardiac death (Lemaitre et al., 2010, 2009). Our study revealed elevated levels of RBC C22:1 n‐9 and C24:1 n‐9 in CKD patients. These fatty acids could contribute the increased cardiovascular risk in ESRD patients. Future studies are warranted to investigate biologic and prognostic properties of n‐9 fatty acids in cardiovascular disease progression in CKD and ESRD.

### 4.3 Saturated fatty acids

Surprisingly, we did detect increases in total C12:0, C14:0, C16:0, and C18:0 plasma levels (besides C20:2 n‐6 and C20:4 n‐6) in response to hemodialysis. Friedman et al. analyzed C18:0/C16:0 fatty acids and observed no change of C18:0, but a decrease of C16:0 fatty acid in response to a single hemodialysis (Friedman et al., 2008). The reasons for these changes and the discrepancies are unknown. The mechanism by which hemodialysis raises levels of individual saturated (C12:0, C14:0, C16:0 and C18:0) and n‐6 (C20:2 n‐6 and C20:4 n‐6) fatty acids in plasma is not known. Since long‐chain PUFA cannot be synthesized endogenously in appreciable amounts, accelerated release into plasma could be a possible explanation. Consistently, the observed accumulation of fatty acids in plasma in did not occur at the expense of free fatty acids. In support of this notion, hemodialysis treatment has been shown to upregulate lipoprotein lipase and phospholipase A2 activity, both of which produce fatty acids from triglycerides and phospholipids, respectively (Friedman et al., 2008; Watkins, Li, Allen, Hoffmann, & Seifert, 2000). The more pronounced changes observed within the blood plasma, as compared with the RBC compartment, are not unexpected since plasma is considered more dynamic with respect to fatty acid flux (Friedman et al., 2008).

## 5 CONCLUSIONS

Our results suggest that hemodialysis treatment does not change the levels of RBC n‐3 fatty acid status of ESRD patients in the systemic circulation. Our study revealed significant differences in total and free RBC fatty acid status between ESRD patients and control subjects, although the omega‐3 quotients did not vary between both groups. We also found that hemodialysis did not induce increased mobilization of individual free fatty acids into plasma or erythrocytes, but caused greater rate of oxidation of total fatty acids, which accumulated (C12:0, C14:0, C16:0, C20:2 n‐6 and C20:4 n‐6) in the circulating blood during hemodialysis treatment. Further studies are needed to elucidate the individual fatty acids altered in CKD for their cardiovascular risk predictions.

## CONFLICT OF INTEREST

None.

## AUTHOR CONTRIBUTIONS

BG, MG, and FCL planned and designed the experimental studies. ID and MR performed the HPLC‐MS spectrometry experiments. All authors contributed to the implementation and analyses of the experiments. BG drafted the article, and all authors contributed to its completion.
